# Trabecular-bone mimicking osteoconductive collagen scaffolds: an optimized 3D printing approach using freeform reversible embedding of suspended hydrogels

**DOI:** 10.1186/s41205-025-00255-0

**Published:** 2025-03-11

**Authors:** Michael G. Kontakis, Marie Moulin, Brittmarie Andersson, Norein Norein, Ayan Samanta, Christina Stelzl, Adam Engberg, Anna Diez-Escudero, Johan Kreuger, Nils P. Hailer

**Affiliations:** 1 OrthoLab, Department of Surgical Sciences/Orthopaedics, Uppsala University, Uppsala, SE-751 85 Sweden; 2 Department of Medical Cell Biology, Science for Life Laboratory, Uppsala University, Uppsala, SE-751 23 Sweden; 3 Department of Chemistry - Ångström Laboratory, Macromolecular Chemistry, Uppsala University, Uppsala, SE-751 21 Sweden

**Keywords:** FRESH, Bioprinting, Additive manufacturing, Tissue engineering, Collagen

## Abstract

**Background:**

Technological constraints limit 3D printing of collagen structures with complex trabecular shapes. However, the Freeform Reversible Embedding of Suspended Hydrogels (FRESH) method may allow for precise 3D printing of porous collagen scaffolds that carry the potential for repairing critical size bone defects.

**Methods:**

Collagen type I scaffolds mimicking trabecular bone were fabricated through FRESH 3D printing and compared either with 2D collagen coatings or with 3D-printed polyethylene glycol diacrylate (PEGDA) scaffolds. The porosity of the printed scaffolds was visualized by confocal microscopy, the surface geometry of the scaffolds was investigated by scanning electron microscopy (SEM), and their mechanical properties were assessed with a rheometer. The osteoconductive properties of the different scaffolds were evaluated for up to four weeks by seeding and propagation of primary human osteoblasts (hOBs) or SaOS-2 cells. Intracellular alkaline phosphatase (ALP) and lactate dehydrogenase (LDH) activities were measured, and cells colonizing scaffolds were stained for osteocalcin (OCN).

**Results:**

The FRESH technique enables printing of constructs at the millimetre scale using highly concentrated collagen, and the creation of stable trabecular structures that can support the growth osteogenic cells. FRESH-printed collagen scaffolds displayed an intricate and fibrous 3D network, as visualized by SEM, whereas the PEGDA scaffolds had a smooth surface. Amplitude sweep analyses revealed that the collagen scaffolds exhibited predominantly elastic behaviour, as indicated by higher storage modulus values relative to loss modulus values, while the degradation rate of collagen scaffolds was greater than PEGDA. The osteoconductive properties of collagen scaffolds were similar to those of PEGDA scaffolds but superior to 2D collagen, as verified by cell culture followed by analysis of ALP/LDH activity and OCN immunostaining.

**Conclusions:**

Our findings suggest that FRESH-printed collagen scaffolds exhibit favourable mechanical, degradation and osteoconductive properties, potentially outperforming synthetic polymers such as PEGDA in bone tissue engineering applications.

**Supplementary Information:**

The online version contains supplementary material available at 10.1186/s41205-025-00255-0.

## Background

Complex fractures, tumors, and infections can cause large bone defects that often require multiple and complex surgeries [1, 2]. Bone tissue engineering involves the creation of resorbable scaffolds that can reduce the burden of multiple surgeries and bone grafting by transferring osteogenic cells together with a suitable carrier to defect sites [3]. The function of a bone scaffold is twofold: first, it bridges the bone defect; and second, it accommodates osteogenic cells that can proliferate, differentiate, and finally transform the scaffold into bone, involving complex interactions between biomaterials and osteogenic and immunomodulatory cells [4]. Synthetic, natural or composite polymers and bioactive ceramics have previously been extensively investigated in the field of bone tissue engineering [5].

Many synthetic polymers are potential candidates for use in bone tissue engineering. Poly(ethylene glycol)-diacrylate (PEGDA) is one such established material in the field, with applications in creating biomimetic scaffolds for bone [6], cartilage [7], and ligaments [8], and is considered non-toxic and of low immunogenicity [9]. PEGDA hydrogels can be crosslinked in the presence of a photoinitiator, with a free radical polymerization reaction between the acrylate functional groups [10]. In the setting of stereolithography (SLA) 3D printing, the addition of a photoabsorber (e.g., tartrazine) can improve the resolution of the printed structures significantly, allowing the creation of multiple complex shapes with high resolution [11]. By adjusting PEGDA’s properties such as the molecular weight and concentration, it becomes possible to optimize the mechanical properties of the construct according to the specific requirements of the application [12]. Polylactic acid (PLA) is another synthetic polymer being investigated as a bone substitute. It offers the advantage of being biodegradable and biocompatible, and it is already used for medical devices [13]. 3D-printed porous PLA scaffolds are thus considered promising substrates for primary osteogenic cell differentiation in vitro [14] and bone formation in vivo [15]. However, clinical experience with synthetic polymers is not unambiguously positive. For instance, PLA-based resorbable screws are considered inferior to their conventional metallic counterparts [16, 17]. PLA- and PEGDA-based scaffolds also lack bioactive surface epitopes, resulting in poor cell attachment [13, 18].

On the other hand, natural polymers such as collagen, cellulose, silk fibroin, alginate, chitosan, and starch may be superior to synthetic polymers for applications in bone tissue engineering due to their biological properties [5, 19, 20]. Type I collagen can in this context be considered an ideal candidate material, particularly due to its abundance in the organic phase of the human skeleton, as well as its exceptional biocompatibility, biodegradation and binding capacity to receptors of the cell membrane [21, 22]. There are different ways to fabricate collagen or collagen-composite scaffolds, including electrospinning [23], compression molding [24], freeze-drying [25, 26], the immersion method [27], and layer-by-layer solvent casting [28]. However, compared with these fabrication methods that mostly render fairly simple 3D geometries, recent advances in 3D printing may offer greater control over the final geometry of the construct [29, 30]. A ground-breaking 3D printing approach was described by Lee et al. [30], making it possible to generate complex and porous collagen scaffolds with a resolution of up to 10 μm. This technique utilizes the freeform reversible embedding of suspended hydrogels (FRESH) approach, whereby collagen is extruded into a support bath consisting of thermoreversible gelatin microparticles. pH-driven self-assembly of the printed collagen takes place within this bath, and subsequently, the gelatin support can be eliminated by raising the temperature to 37 °C [30]. With the ability to achieve a mean filament diameter of 100 μm [31], FRESH printing allows for precise control over the microarchitecture of scaffolds, particularly in creating porous or trabecular structures using soft hydrogels like collagen. Scaffold porosity is an important determinant for cell adhesion, infiltration, and viability [32, 33] in three-dimensional structures intended for bone regeneration, through increased surface area [34]. Moreover, gelatin microparticles in the FRESH support bath create micro concavities in the printed collagen filament [30, 31], which are likely favorable to promote osteogenic cell attachment.

As an example, the FRESH printing method was previously used to manufacture cell-laden type I collagen scaffolds with a lattice structure for nasal cartilage tissue [35], resulting in a uniformly deposited cartilage extracellular matrix in vitro. However, the osteoconductive properties of FRESH-printed scaffolds have not previously been extensively investigated. Here, our objective was to evaluate the material properties and osteogenic profile of 3D printed trabecular collagen scaffolds manufactured by FRESH printing, and to compare them to scaffolds generated with the established synthetic material PEGDA.

## Methods

### Scaffold fabrication

A cylindrical 3D structure (6 mm × 5 mm, diameter × height) with a solid cortical periphery (1 mm thick) and a trabecular porous configuration at its center was designed using Netfabb software (Autodesk) and used for printing the collagen and PEGDA scaffolds. The trabecular geometry generated had an anisotropic porous structure with an average filament size of 100 μm and average pore size of 300 μm.

The trabecular bone design was exported as an STL file and sliced in the PreForm software (Formlabs) for SLA printing. The printing settings used in the PreForm slicing software for the SLA printing process are included in a supplementary text file (Supplementary Material 1). For FRESH printing, the STL file was sliced in the Simplify3D software. The printing settings used in the Simplify3D slicing software (Simplify3D, Version 4.1.2) are included in a supplementary text file (Supplementary Material 2).

The PEGDA scaffolds were fabricated using a modified Form 1 + SLA printer (Formlabs, Somerville, MA, USA) (Supplementary Fig. 1A, B). The build platform consisted of a microscopy glass (75 mm × 25 mm × 1 mm) glued on a support attached to the Z-axis of the printer and a Petri dish served as PEGDA solution tank. Prior to its attachment, the microscopy glass was immersed in a bath with a binding-silane for 10 min; it was then rinsed with ethanol 95% and placed in a container immersed in a water bath set to 100 °C for 10 min to increase PEGDA scaffold attachment. The surface of the Petri dish was covered with 10 mL of polydimethylsiloxane (PDMS; Dow Corning Sylgard 184, Silmid, Galindberg Sweden) to make it hydrophobic and avoid inadvertent attachment of the PEGDA scaffolds. After the PDMS layer was applied, the Petri dish was placed under a vacuum for 25 min to remove any trapped air bubbles and then cured in an oven at 80 °C for 45 min. The solution used for printing consisted of 20% w/w PEGDA (molecular weight, 700 Da), 0.5% w/w lithium phenyl-2,4,6-trimethylbenzoylphosphinate (LAP) (the photoinitiator), 0.19% w/w tartrazine photoabsorber (all from Sigma-Aldrich, Stockholm, Sweden), and deionized water to a total weight of 25 g. The PEGDA solution was poured onto the hydrophobic PDMS-coated Petri dish. The scaffolds were then printed with a 405 nm light source, with the (x, y) printing positions aligned to the location of the microscopy glass used as the build platform. After printing the PEGDA scaffolds were cured for 5 min under UV light using a curing machine (Curator M, 3DVerkstan AB, Sweden).

Collagen scaffolds were printed using the FRESH technique [30, 36] and an open source extrusion bioprinter based on the E3D motion system and tool changer, featuring a transparent polycarbonate cabinet with an integrated HEPA filter and air intake fan to enable contamination-free 3D printing (Supplementary Fig. 1C, D) [31]. After initial FRESH printing tests, it was observed that the collagen scaffolds were 3% larger than the corresponding computer-aided design (CAD) file due to swelling. A 3% reduction was therefore subsequently applied in the slicing software to correct for this. This adjustment allowed for the generation of collagen scaffolds similar in size to the PEGDA scaffolds.

Bovine acid solubilized collagen type I (Lifeink 240 Collagen Bioink, Acidic pH, Cellink) at a concentration of 35 mg mL^− 1^ was printed into a gelatin support bath at neutral pH using a glass Hamilton syringe (250 µL), fitted with a 27G needle (0.21 mm × 12.7 mm, internal diameter × length) at a printing speed of 10 mm s^− 1^. The gelatin support bath was prepared in sterile conditions by hydration of FRESH LifeSupport Powder (Cellink) with the addition of cold phosphate-buffered saline (PBS, 1×) according to the manufacturer’s instructions. Printing was performed in a custom-made 3D-printed polyetherimide (PEI) basket, designed to fit into the wells of a 12-well plate, to allow for easier handling of the scaffolds. After printing, the collagen scaffolds embedded in the gelatin support rambath were placed at 37 °C for 30 min to further cross-link the collagen and to liquify the gelatin support. The PEI baskets containing the collagen scaffolds were subsequently lifted out of the melted gelatin and washed three times in 1× PBS at 37 °C.

The PEGDA and collagen scaffolds were finally moved into a 96-well plate for sterilization by immersion in 70% ethanol for 20 min and were then rinsed three times with PBS. The scaffolds were kept in 96-well plates for further experimentation.

### Scaffold characterization

#### Confocal microscopy and scanning electron microscopy (SEM)

Scaffold porosity was visualized with confocal microscopy after staining the structures with fluorescent dyes. Entire collagen scaffolds were fixed with 4% v/v paraformaldehyde at room temperature for 20 min, rinsed 3 times with PBS, stained with Sirius red 0.1% in saturated picric acid (HistoLab Products AB, Sweden) for 1 h at room temperature, rinsed twice with 0.5% acetic acid in water, and left in water overnight before imaging. PEGDA structures consisting of the first 4 layers of the original sliced CAD model were printed, stained with TRITC (Tetramethylrhodamine isothiocyanate) for 5 min at room temperature, and rinsed twice with water before imaging. To capture the entire surface of the scaffold and the thickness corresponding to one printed layer, a tile scan z-stack module was used with a Plan-Apochromat 20×/0.8 (Zeiss) objective on a Laser Scanning Microscope (Zeiss). Images were processed as z-projection in Image J and used for porosity visualization over one printed layer of the PEGDA and collagen scaffold. Single slices or z-projection images from two independent collagen and two independent PEGDA prints were used to evaluate filament diameter and porosity. Filament diameter was measured in 8 different locations for each image, using the line tool on Image J. Porosity was measured in the porous central area (not including the cortical periphery) by creating a selection and setting the threshold on the images to ensure inclusion of all the fluorophore stained collagen or PEGDA structures. Porosity was obtained as a porosity% / area and expressed as a porosity% / mm^2^ by considering the area of the created selection used to measure porosity in the central trabecular area. To obtain the porosity of one printed layer from the original PEGDA and collagen CAD model, two images of a layer of the thickness of one printed layer during the SLA (55 μm) or FRESH (100 μm) printed process were extracted and analyzed using the same method. These two images of the CAD model correspond to the first and last printed layer of the scaffolds.

The surface morphology of PEGDA and collagen scaffolds without cells were imaged using a scanning electron microscope (SEM; Zeiss LEO 1550 with Oxford AZtec EDS) with a high-resolution secondary electron InLens detector at an accelerating voltage of 2 kV. PEDGA scaffolds were dried by freeze-drying and collagen scaffolds by critical point drying. Any PBS present in the PEGDA scaffolds was replaced by water before freeze-drying to avoid the formation of salt precipitates. Collagen scaffolds were dehydrated using the following dehydration series: 20% ethanol, 40% ethanol, 60% ethanol, 80% ethanol, absolute ethanol, 2/3 absolute ethanol/1/3 absolute acetone, 1/3 absolute ethanol/2/3 absolute acetone, and then rinsed in absolute acetone. Collagen scaffolds were immersed in the different baths for 15 min at room temperature. Acetone was then replaced by liquid CO_2_, which in turn was evaporated at the critical drying point of CO_2_ (31 °C, 1072 psi) to preserve the sensitive collagen scaffolds using a Polaron E3000 (Agar Scientific, Stansted, UK) critical point dryer. The PEGDA and collagen scaffolds were placed on a stub with carbon tape and coated with 10 nm of Au/Pd using a sputter coater (Polaron SC764).

#### Rheological properties

Rheology was performed using a Discovery Hybrid Rheometer 2 (DHR2, TA instruments, Sollentuna, Sweden). Amplitude sweeps were performed at a constant frequency of 0.1 Hz by varying the torque from 0.01 to 1.0 µN m. An 8-mm parallel plate stainless steel geometry was used. The diameter of the printed scaffolds was 6 mm, therefore the geometry diameter was modified to 6 mm in the instrument software to obtain a more accurate calculation of the oscillation stress. Eight mm is the smallest diameter available for parallel plate geometry from TA Instruments. Samples were placed centrally by drawing a circle with a diameter of 8 mm on the bottom of the rheometer plate and the tests were initiated once an axial force of 0.03 N was recorded. The samples were observed constantly during the measurement to ensure that no sidewise slip occurred. The storage (G′) and loss (Gʺ) modulus of the printed scaffolds were extracted.

#### Mechanical properties

3D-printed type I collagen scaffolds were tested for their compression properties on a Discovery Hybrid Rheometer 2 (DHR2, TA instruments, Sollentuna, Sweden) using a 40-mm parallel plate stainless steel geometry. DHR2 has a maximum normal force limit of 50 N with a sensitivity of 0.005 N and resolution of 0.5 N. First, the gap between the upper and lower plate was calibrated without the sample using the default instrument parameters, so that when a normal force of 5 N is achieved, the instrument sets a gap of 0 μm. Printed scaffolds were placed centrally onto the rheometer plate with lubrication on both surfaces by placing one drop of PBS to prevent barreling during compression. The top plate was lowered until it touched the sample surface as observed visually and by obtaining a normal force of 0.01 N. The normal force was tared to zero immediately before starting the experiment. The head speed was maintained at 5000 μm min^− 1^, and four scaffolds were compressed once to a strain value of 30%. Engineering stress was calculated using the sample diameters at the start of the experiment (cylindrical shape with 6 mm diameter and 5 mm height). For compression, no shear was applied and only axial head movement of the rheometer was used.

#### Degradation

The degradation properties of the PEGDA and collagen scaffolds were evaluated by measuring their initial dry mass after printing, and the dry mass after 4 weeks in PBS supplemented with 1% of penicillin/streptomycin at 37 °C and 5% CO_2_. Three samples per time point were freeze-dried using liquid nitrogen prior to weighing.

### Biological characterization

#### Cell isolation

In this study, we used passages 4–6 of primary human osteoblasts (hOBs) from patients treated at the arthroplasty service of the University Hospital of Uppsala, and passages 11–15 of pre-osteoblastic human osteosarcoma cell lines (SaOS-2, ECACC, Sigma-Aldrich, UK). The hOBs were isolated from femoral heads from patients who underwent hip arthroplasty according to a previously published protocol [37]. The femoral heads were diced into small fragments, which were rinsed with PBS and then placed in 25-cm^2^ flasks containing alpha modified minimum essential medium (αMEM; Merck KGaA, Darmstadt, Germany), 10% fetal bovine serum (FBS; Merck KGaA), 1% penicillin/streptomycin, and 0.5% amphotericin. The culture medium was refreshed once per week.

As a control experiment, the cells were cultured two-dimensionally (2D) in 96-well plates in collagen-coated wells or non-coated wells. The cells were cultured three-dimensionally (3D) on 3D-printed collagen or PEGDA scaffolds. For the 2D cultures, type I rat tail collagen 3.32 mg mL^− 1^ (90% purity; Corning, 354236) was used for coating the wells, supplemented with 8% Dulbecco’s modified essential medium (DMEM; Merck KGaA) and 0.5% NaHCO_3_; 100 µL of this solution was placed in every well and then aspirated back. The 96-well plates were then left in the incubator (37 °C, 5% CO_2_) for 2 h. After sterilization, the scaffolds were incubated overnight (37 °C, 5% CO_2_) with 100 µL of cell medium per well, consisting of DMEM or αMEM, with 10% FBS, 1% penicillin/streptomycin, and 0.5% amphotericin. The next day, 10^4^ cells were seeded in the wells and on the scaffolds in 20-µL droplets; 80 µL of cell medium was added to every well, to a complete volume of 100 µL. The medium was exchanged two times per week. The medium was enriched after the first week of culture with 10 mM β-glycerophosphate, 100 nM dexamethasone, and 80 µM ascorbic acid (Merck) to further stimulate osteogenic activity, and the cultures were subsequently grown for three additional weeks.

#### Cell osteoblastic activity

Osteoblast activity was assessed using the alkaline phosphatase (ALP) colorimetric assay at 1, 2, and 4 weeks of culture. ALP activity, associated with bone formation and mineralization, was quantified and normalized against lactate dehydrogenase (LDH) activity, serving as a proxy for the number of cells. For each measurement the medium was removed from the 96-well plates and the cells and scaffolds were rinsed twice with PBS. Next, the cells were lysed enzymatically in their wells with 150 µL of lysis buffer (sodium CellLytic; Sigma-Aldrich, Sweden) for 15 min on a shaker at 300 rpm at room temperature. Then, 50 µL of cell lysate was analyzed for LDH activity as per the manufacturer’s protocol (LDH, TOX7; Merck KGaA), and the absorbance was measured in a spectrophotometer at 690 and 492 nm (Multiscan Ascent, Thermo Fisher Scientific, Waltham, MA, USA) after 30 min in the dark at room temperature. The absorbance at 690 nm was subtracted from the absorbance at 492 nm. ALP activity was quantified in a similar fashion; 50 µL of the lysate was mixed with the ALP substrate (*p*-nitrophenyl phosphate; Merck KGaA) and then the absorbance was measured at 405 nm after 30 min in an incubator (37 °C, 5% CO_2_). Osteoblast activity was expressed as the mean ALP/LDH ratio (± standard deviation). In the 2D setting, there were 4 biological replicas in collagen-coated wells and 7 replicas for non-coated wells. In the 3D setting, there were two biological replicas for SaOS-2 cells on PEGDA and collagen scaffolds, 4 biological replicas with hOBs on PEGDA, and three on collagen scaffolds. Triple technical replicates were performed.

#### Cell morphology

The morphology of the cells seeded in the wells and on the collagen scaffolds was visualized by confocal laser scanning microscopy using a Carl Zeiss LSM 700 Laser Scanning Microscope (Carl Zeiss) after 1, 2, and 4 weeks of culture. SaOS-2 seeded on the PEGDA scaffolds were imaged with the same microscope. Cell nuclei were stained with 4′,6-diamidino- 2-phenylindole (DAPI; Invitrogen, Waltham, MA, USA) and the cytoplasm with carboxyfluorescein diacetate (CFDA; Merck KGaA). Intracellular osteocalcin (OCN) was detected with immunofluorescence.

The cells were fixed with 4% v/v paraformaldehyde at room temperature for 20 min, rinsed three times with PBS, and permeabilized using 0.1% Triton X-100 (Merck KGaA) for 15 min. The cytoplasm was stained with CFDA (500 nM) for 15 min, then normal 10% goat serum (s-1000; Sigma-Aldrich, Sweden) blocking solution in PBS with 2% bovine serum albumin (BSA) and 0.3% Triton X-100 for 30 min. The anti-OCN antibody (20 µg mL^− 1^ human/rat OCN, MAB1419; R&D Systems, Abingdon, UK) diluted in PBS/2% BSA/0.3% Triton X-100 was added subsequently and the plates were incubated overnight at 4 °C. The wells were rinsed four times with PBS/1% Triton X-100, the secondary antibody (1:200, goat anti-mouse, Biotin Novus NB7537; Bio-Techne, Abingdon, UK) was added, and the wells were left under agitation at room temperature Non-specific binding of secondary antibody was controlled in 2D by omitting the primary antibody in a control well. The wells were then rinsed four times with PBS/1% Triton X-100 and stained with DAPI (300 nM) and Dylight Streptavidin red (Vector sa-5549, concentration 20 µg mL^− 1^), both dissolved in PBS for 30 min at room temperature and then rinsed four times with PBS/1% Triton X-100. After the staining procedure and washing, the collagen scaffolds were extracted from the 96-well plate using a micropipette and a 1-mL pipette tip cut to encompass the cylindrical scaffold without damaging it during extraction. PEGDA scaffolds were extracted from the wells using needles. The side of the scaffolds that was seeded with cells was placed face down in a dish with a glass coverslip bottom (ibidi, Munich, Germany) for imaging, keeping it hydrated with PBS.

Images were obtained using a Plan-Apochromat 10×/0.45 (Zeiss) objective. Cells present in the collagen scaffolds were imaged using 14-µm sections along the Z axis. The Z stacks were post-processed in maximal intensity projection in Image J. The images were taken by keeping the same gain for all wells and all time points within a replicate.

### Statistics

Statistical tests and graphs were performed with R version 4.3.3 [38], with a level of significance of *p* < 0.05. Levene’s test was performed to asses if the assumption of homogeneity of variance was met. One-way ANOVA was performed, followed by Turkey’s post hoc test to identify differences between groups. The Kruskal-Wallis test was used if homogeneity of variances was not met, along with Dunn’s (1964) post-hoc multiple comparison test with a Bonferroni correction applied. Student’s t test was used to compare mean values between two groups.

## Results

### Generation of collagen and PEGDA scaffolds

**Fig. 1:**
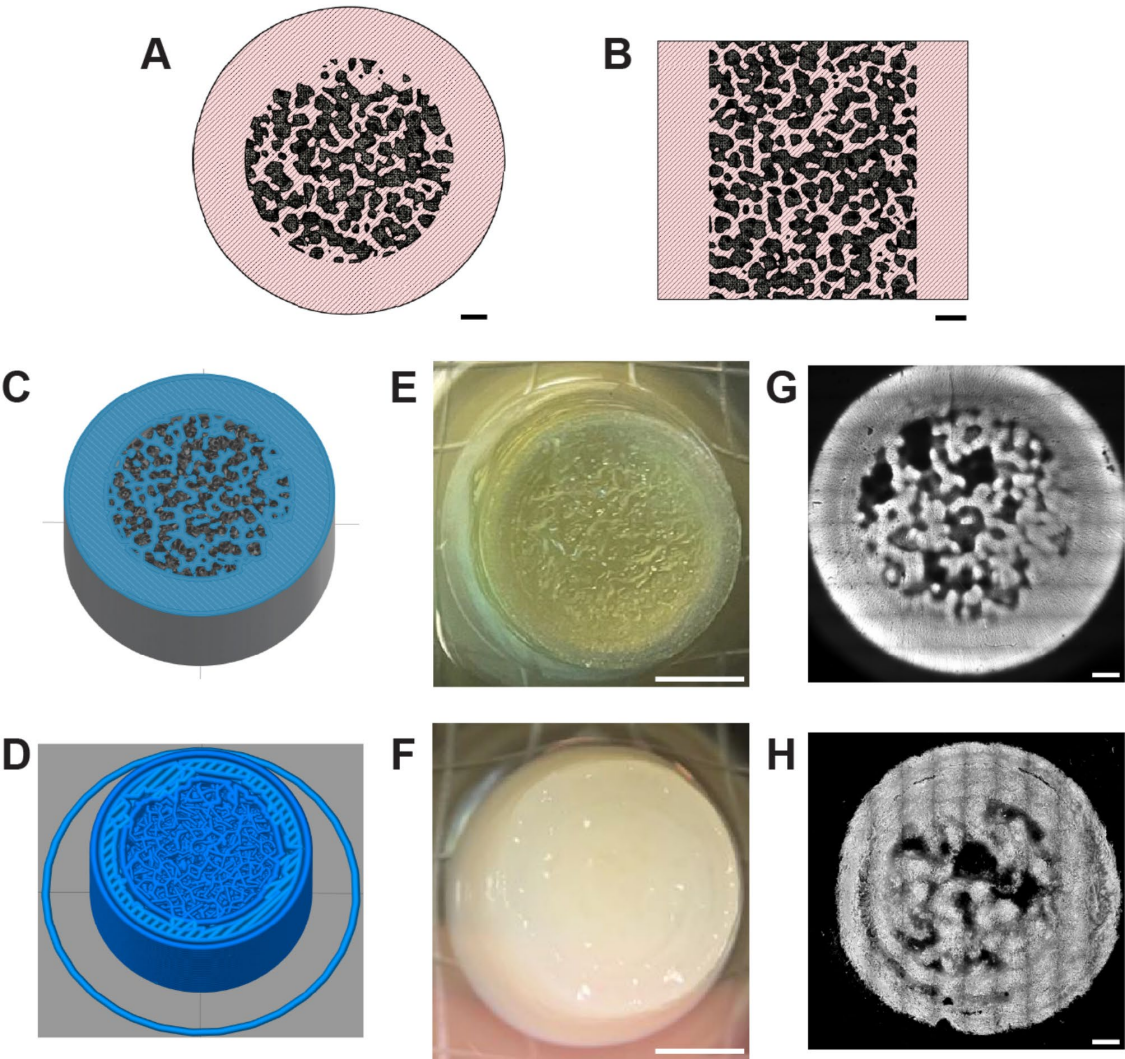
Trabecular bone-mimicking scaffolds fabricated with 3D printing techniques using PEGDA or type I collagen. Top view (**A**) and side view (**B**) of the CAD model used for 3D printing of PEGDA and collagen scaffolds. (**C**, **E**, **G**) PEGDA scaffolds. (**D**, **F**, **H**) Collagen scaffolds. The trabecular model was sliced using either the PreForm software for SLA printing (**C**) or the Simplify3D software for FRESH printing (**D**). A cross-sectional view of the middle of the model is shown. Top view of the printed PEGDA scaffold (**E**) and printed collagen scaffold (**F**). Porosity of the PEGDA (**G**) and collagen (**H**) scaffolds as captured by confocal microscopy. Z projection images corresponding to one printed layer of the PEGDA structure stained with TRITC (Tetramethylrhodamine isothiocyanate) and one printed layer of the collagen scaffold stained with Picrosirius red. Scale bars: 500 μm (**A**, **B**, **G** and **H**), 2 mm (**E** and **F**)

The FRESH 3D printing and SLA 3D printing techniques were used to fabricate scaffolds using an identical computer-aided design (CAD) file (Fig. 1A, B), which was designed to replicate a trabecular bone structure surrounded by a solid cortical periphery. The sliced trabecular models (Fig. 1C, D) capture the porosity of the CAD design, which led to the successful printing of PEGDA scaffolds (Fig. 1E) and collagen type I scaffolds (Fig. 1F) with trabecular-like porous architecture (Fig. 1G, H). Both scaffold types demonstrated structural stability and were easy to handle using a spatula. The collagen type I scaffolds had a soft texture, whereas the PEGDA scaffolds were more rigid.

Confocal microscopy of the scaffolds stained with fluorescent dyes allowed to visualize porosity of one representative printed layer of the PEGDA (Fig. 1G) and collagen (Fig. 1H) scaffolds. The PEGDA and collagen scaffolds showed different porous architecture likely influenced by the differences in resolution between the SLA and FRESH printing techniques. Filament diameter and porosity were measured on two independent images of one printed PEGDA layer and two independent images of one printed collagen layer (Table 1). Filament diameter was measure in 8 different locations in each image. Porosity was measured in the central porous area of the images of the structures from two independent prints for both collagen and PEGDA scaffolds. The area of the central porous part of the scaffold of each scaffold was used to express porosity as a % / mm^2^. These results were compared with expected filament diameter and measured porosity from one printed layer of the original STL files of the PEGDA and collagen scaffolds. The PEGDA scaffolds present a filament diameter more consistent to the STL file generated from the CAD design. Indeed, during FRESH printing a 210 μm internal diameter needle was used, decreasing printing resolution of the original CAD file designed with an average filament diameter of 100 μm. Porosity of the printed scaffolds was inferior to intended porosity.

**Table 1:** Filament diameter (µm) and porosity (% / mm^2^) were measured from images corresponding to one printed layer of the CAD model of the PEGDA and collagen scaffolds, and one printed layer of the PEGDA and collagen scaffolds

Measurements	CAD model PEGDA trabeculae	Printed PEGDA filament	CAD model collagen trabeculae	Printed collagen filament
Diameter (µm)	∼ 100	176.8 ± 18.8	∼ 100	250.0 ± 44.6
Porosity (%/mm^2^)	∼ 4.2	∼ 3.5	∼ 3.5	∼ 3.0

### Analysis of collagen and PEGDA scaffolds by scanning electron microscopy

**Fig. 2:**
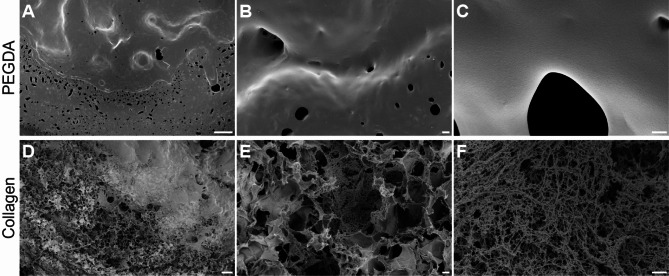
Scanning electron microscopy imaging of 3D-printed PEGDA and collagen scaffolds. Samples of PEDGA scaffolds (**A**–**C**) were freeze-dried, and samples of FRESH-printed collagen scaffolds (**D**–**F**) were prepared using ethanol and acetone dehydration followed by critical point drying. Scale bars: 100 μm (**A** and **D**), 2 μm (**B**, **C**, **E**, and **F**)

Scanning electron microscopy (SEM) imaging was performed to analyze the surface morphology of the PEGDA and collagen scaffolds (Fig. 2). The SEM images showed distinctly different morphologies; the PEGDA scaffolds had very smooth surfaces, whereas the collagen scaffolds were characterized by an intricate and fibrous 3D network of collagen fibrils with a diameter of approximately 70 nm.

### Viscoelastic and degradation properties of collagen scaffolds

**Fig. 3:**
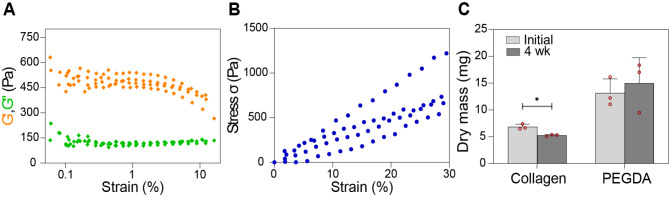
Rheological, mechanical and degradation characterization of 3D-printed collagen scaffolds. Storage (G′) and loss (Gʺ) modulus of collagen scaffolds were determined from an amplitude sweep at 0.1 Hz, at a strain of 1% (**A**). The compressive Young’s modulus of collagen scaffolds was determined using an unconfined static compression at a strain rate of 5000 μm min^− 1^ (**B**). Tests were performed on four different scaffolds. The mass changes of the collagen scaffolds and PEGDA scaffolds (**C**) were determined by weighting the dry mass of the scaffolds after printing and after 4 weeks in PBS; *p < 0.05

Next, we conducted an amplitude sweep analysis to investigate the viscoelastic properties of 3D-printed collagen scaffolds. The storage modulus (G′) was consistently higher than the loss modulus (Gʺ) for all investigated scaffolds, indicating a predominantly elastic response rather than viscous behavior (Fig. 3A). At a strain of 1%, the average storage modulus (G′) was 492.2 ± 30.9 Pa, and the average loss modulus (Gʺ) was 115.6 ± 7.6 Pa. These values are in the range of bulk collagen hydrogels, with lower concentrations reported in the literature, but considerably lower than in collagen hydrogels modified by chemical crosslinking [39–41]. The porosity of the collagen scaffolds and physical self-assembly by pH neutralization tend to make the scaffolds more flexible, lowering the storage modulus.

The loss tangent (tanδ) was 0.23, indicating a moderate damping effect suggestive of some degree of energy dissipation within the ranges of 0.5– 3% strain. Further, we measured a compressive Young’s modulus of 2.7 ± 0.9 kPa for the collagen scaffolds, using the stress–strain curve (Fig. 3B) obtained from the unconfined compression test. This value is lower than previously reported values for bulk collagen gels [42].

Finally, we investigated the degradation properties of the collagen scaffolds (Fig. 3C) by measuring the dry mass of the scaffolds after printing and after 4 weeks of storage in phosphate-buffered saline PBS. The collagen scaffolds with an initial mass of 6.84 ± 0.5 mg, showed a significant mass loss of an average 1.59 ± 0.5 mg after 4 weeks, in contrast to the PEGDA scaffolds which showed no degradation over the same time period.

### Osteoblastic activity of cells grown in 2D and 3D on PEGDA and collagen scaffolds

**Fig. 4:**
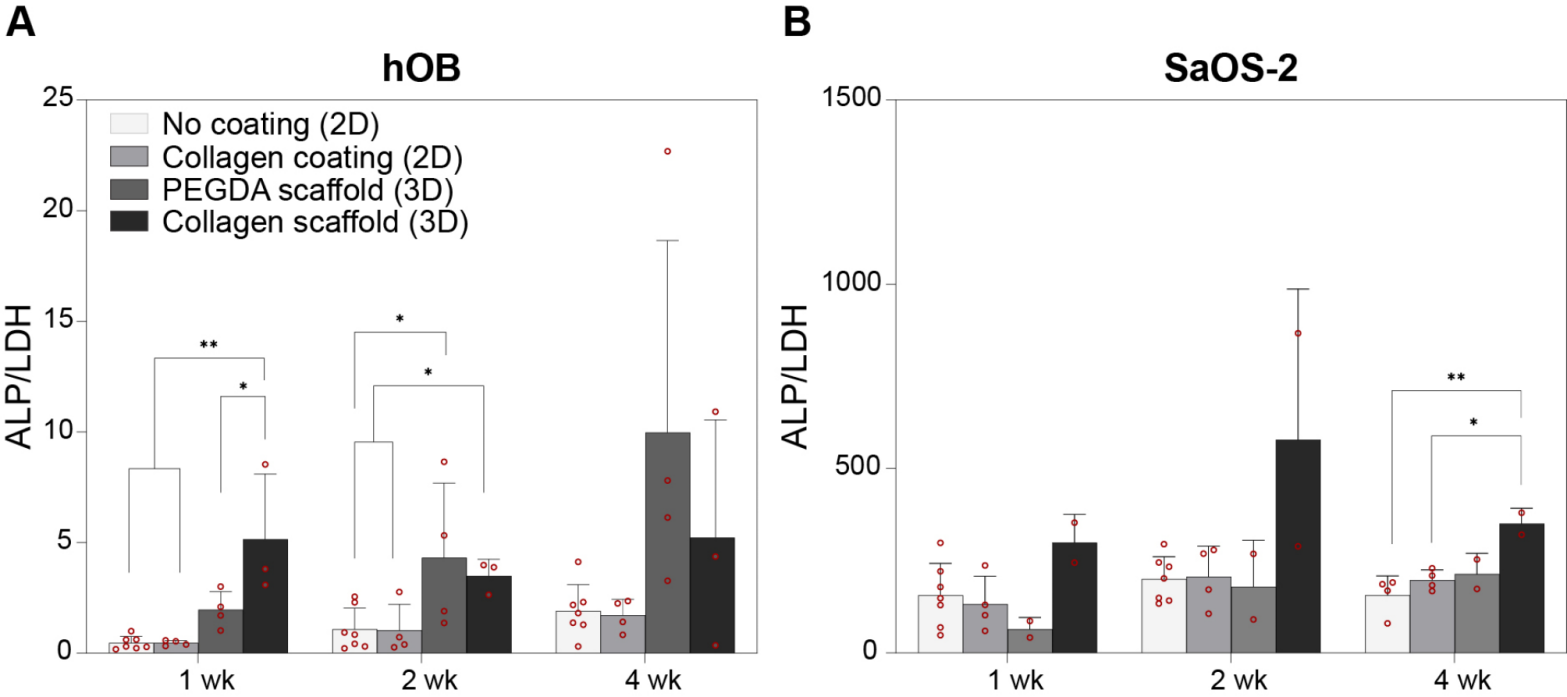
Osteoblastic activity of hOBs and SaOS-2 cells grown on 2D or on 3D-printed collagen, or on PEGDA scaffolds, assessed by ALP and LDH activity measurement. ALP activity normalized by LDH activity in hOBs (**A**) and SaOS-2 cells (**B**) grown in 2D in non-coated wells or in collagen-coated wells, and in 3D on PEGDA and collagen scaffolds. hOBs, human osteoblasts; ALP, alkaline phosphatase; LDH, lactate dehydrogenase; *p < 0.05, **p < 0.005

For both hOBs and SaOS-2, the collagen coating (Supplementary Fig. 2B) did not affect their osteogenic activity as there were no statistical differences measured in ALP activity between the uncoated and collagen-coated groups for each time point (Fig. 4A, B). However, hOBs showed increased ALP activity when cultured on PEGDA and collagen scaffolds (3D) compared to 2D cultures at each time point. At weeks 1 and 2, statistically significant differences were observed between 2D and 3D cultures, while the ALP/LDH ratio was higher for the cells that were grown on collagen scaffolds versus PEGDA at week 1 (Fig. 4A). SaOS-2 cells exhibited a trend for higher ALP activity in collagen scaffolds (3D) compared to 2D cultures and PEDGA scaffolds (Fig. 4B); that difference was statistically significant at week 4 between 2D cultures and collagen scaffolds (3D).

The ALP activity of bone cells grown on FRESH-printed collagen scaffolds was similar to the activity of cells grown on PEGDA scaffolds (Fig. 4A, B). ALP activity stayed fairly constant over the 4 weeks of culture in the 3D constructs, with higher values for SaOS-2 cells.

### Imaging of bone cells grown on collagen and PEGDA scaffolds

**Fig. 5:**
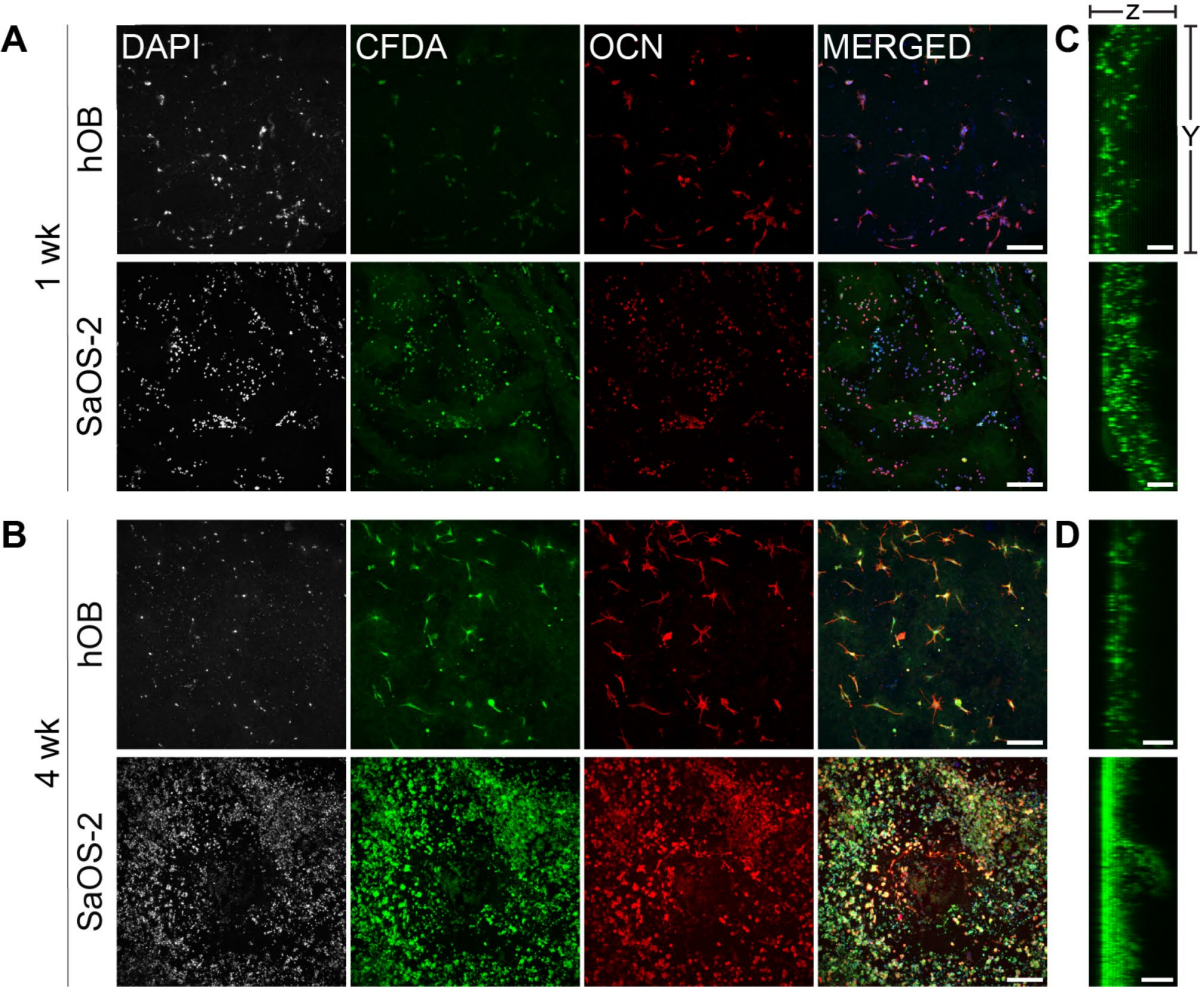
Immunocytochemistry of hOBs and SaOS-2 cells grown on FRESH-printed collagen scaffolds. Maximum intensity z projection images captured by confocal microscopy of hOBs and SaOS-2 cells grown on collagen scaffolds for 1 week (**A**) or 4 weeks (**B**), and stained for cell nuclei (DAPI), cytosol (carboxyfluorescein diacetate [CFDA]), and the osteoblast marker osteocalcin (OCN). hOBs and SaOS-distribution along the z axis after 1 week (**C**) or 4 weeks (**D**). (**C-D**) Images obtained from maximal intensity x projections using the CFDA channel. Scale bars: 200 μm (**A-B**), 100 μm (**C-D**). Both hOBs and SaOS-2 cells colonized the scaffolds within a 4-week timeframe. hOB, human osteoblast

Using confocal microscopy, we visualized osteogenic cells cultured on collagen scaffolds (Fig. 5) as well as collagen-coated wells (Supplementary Fig. 2). SaOS-2 were also imaged on the PEGDA scaffold (Supplementary Fig. 2). The presence and distribution of cells was assessed using staining against osteocalcin (OCN), a protein expressed by osteoblasts during bone formation, together with staining for cell nuclei (DAPI) and labeling of the cytoplasm with CFDA. Confocal imaging was performed at different depths of the collagen scaffolds, allowing to combine the image stacks using maximal intensity projection to create 2D composite images of the scaffolds along the z axis (Figure A, B) and x axis (Fig. 5C, D). hOBs featured a more uniform growth on the surface of the scaffold over a 4-week culture period, whereas, expectedly, SaOS-2 cells demonstrated a more marked proliferation within the collagen structure (Fig. 5A, B). Cell distribution along the z-axis is shown in Fig. 5C and D, illustrating cell penetration at depth of the scaffold after 1 and 4 weeks.

## Discussion

### Main findings

In this study, two distinct hydrogels were utilized for the additive manufacturing of scaffolds that feature intricate geometries. By employing FRESH printing of collagen and SLA of photocrosslinkable PEGDA, structures closely resembling the trabeculae of cancellous bone were successfully printed. We found that the FRESH-printed collagen scaffolds exhibited a microporous structure and viscoelastic mechanical behavior, degraded over time, and were biocompatible, supporting osteogenic activity and colonization by different osteogenic cells.

The rheologic characterization and unconfined compression tests revealed that the storage modulus and compressive Young’s modulus of FRESH-printed collagen scaffolds were lower than previously reported values for collagen gels [42, 43]. This implies that both the shear characteristics and the compressive modulus of the scaffolds are affected by the 3D-printed structure design. The degree of layer-to-layer adhesion and any gaps inside these layer interfaces might have a major impact on the scaffolds’ reaction to stress. In addition, in contrast to denser collagen gels, the scaffolds’ interconnecting pores can promote higher fluid flow, shear strain dispersion, and weakening of the scaffold, resulting in a reduced storage modulus and compressive Young’s modulus. A number of factors, such as collagen type, concentration, self-assembly strategy, and testing conditions, affect the shear and compressive properties of collagen gels. Therefore, caution is advised when comparing the shear and compressive properties of 3D-printed collagen scaffolds produced in the current study with previous results obtained for less complex structure made of collagen. The collagen scaffolds fabricated through FRESH printing had good handling properties, although they were soft, with a Young’s modulus of 2.7 kPa, similar to values previously reported in the literature [44]. This value is significantly lower than the Young’s modulus of cancellous bone that is 3.7 GPa, and the Young’s modulus for load-sharing implants such as PEEK and titanium is 3.8 and 50 GPa respectively [45]. Collagen exhibited a faster degradation rate in comparison to PEGDA, which could be helpful in the clinical setting, where the bone defect heals as the scaffold is resorbed. In the case of PEGDA, Chen et al. [46] proposed the decreasing the percentage of weight of PEGDA by copolymerization with poly(glycerol sebacate) acrylate in order to increase degradation rate, as PEGDA exhibits a much lower degradation rate than collagen.

The osteoblastic activity of hOBs and SaOS-2 cells cultured on collagen scaffolds was higher compared to both uncoated and collagen-coated plastic wells, yet similar to those of PEGDA scaffolds. ALP activity was maintained throughout the 4 weeks of culture. ALP activity in SaOS-2 cells were higher than in hOBs, likely due to the mature osteoblast phenotype of SaOS-2 associated with high levels of ALP activity as previously reported [47]. The different phenotypes of hOBs and SaOS-2 were also evident from the confocal microscopy images of collagen scaffolds; SaOS-2 cells grew faster and formed dense colonies, whereas hOBs exhibited a more scattered pattern after 4 weeks of culture. Both cell types spread on the surface of the collagen scaffolds, and the scaffold porosity allowed for cellular infiltration (Fig. 5). In contrast, fluorescent confocal imaging demonstrated that PEGDA did not appear to be as easily colonizable by SaOS-2 cells at week 4 (Supplementary Fig. 2A); this might be attributed to the material’s poor cellular adhesion properties [48, 49] as well as surface topography properties (Fig. 2). Compared to cells seeded on collagen scaffolds, hOBs grown on collagen-coated wells (Supplementary Fig. 2B) formed a dense cell layer while SaOS-2 cells grown on collagen-coated wells formed cell aggregates. Osteocalcin (OCN), a non-collagenous protein indicating osteoblast maturation, was observed in both hOBs and SaOS-2 grown on collagen scaffolds at weeks 1 and 4 of culture, supporting the fact these scaffolds represent an appropriate substrate to support osteogenic activity [50]. Taken together, these findings encourage further investigation of complex 3D printed collagen structures as potential substrates for bone regeneration in accordance with previous literature [51–54].

### Strengths and limitations

Conventionally, harder materials such as calcium phosphate cements have been considered more suitable for bone tissue engineering, and softer materials such as polyethylene glycol and hydrogels have been preferred for applications involving softer tissues, for example cartilage tissue engineering [55]. With improvements in fixation techniques that can maintain the integrity of a soft graft within a bone defect area, it may be possible to use softer materials in the treatment of bone defects, and given the slow degradation of some ceramic scaffolds, hydrogel-based scaffolds may be more suitable in a clinical context. This bone defect filling approach mimics the intramembranous ossification process that is clinically used during the second stage of the Masquelet procedure [56]. In our study, under the same experimental conditions, we sought to explore the osteogenic properties of trabecular scaffolds generated using different 3D printing techniques, using a synthetic and a natural polymer. To our knowledge, this is the first example of using FRESH printing to engineer osteoconductive scaffolds intended for bone regeneration. After its optimization in 2019 by Lee et al. [30], the FRESH technique enabled the 3D printing of complex collagen scaffolds, ranging from artery- to organ-scale with microscale porosity. In the present study, we engineered a collagen scaffold featuring both trabecular and solid structures, resulting in a stable porous structure with favorable osteogenic activity. Unexpectedly, ALP activity, which measures osteoblastic activity, was not consistently higher in collagen scaffolds compared to scaffolds printed with PEGDA. This was surprising given PEGDA’s lack of surface epitopes, which is otherwise combined with inorganic components or other synthetic polymers to enhance its osteoconductive properties [18, 57]. Finally, the measurement of the osteoconductive properties of the 3D printed scaffold was a major challenge. We intended to complement the study with an evaluation of scaffold mineralization by the osteogenic cells, however the use of Alizarin red to stain calcium deposit secreted by cells was inconclusive due to considerable dye retention within the scaffolds. Furthermore, the absence of an in vivo model limited the extent to which clinical perspectives on these scaffolds could be drawn.

### Perspectives

Additional studies should be conducted to improve the mechanical properties of the 3D-printed collagen scaffolds, by changing the collagen concentration, by applying different cross-linking strategies, or by using more complex bioinks by combining collagen with an inorganic phase such as hydroxyapatite or bioglass [43], or with another organic polymer such as silk fibroin [58]. Other potential strategies to create more mechanically robust collagen-based scaffolds include post-printing modifications such as freeze-drying, UV-irradiation, or dehydrothermal treatment [59]. Modification of the bioink composition could also be of interest to increase the osteoconductive properties of the collagen scaffold by for example including of bone morphogenetic protein (BMPs), transforming growth factor-β (TGF- β), or nanohydroxyapatite particles [52]. In future studies, the experimental setup could be strengthened to allow compatibility with FRESH and SLA bioprinting, allowing for direct inclusion of cells within the 3D scaffolds instead of seeding them post printing. In this study, the low pH of the collagen ink employed for FRESH printing prevented mixing of cells with the ink prior to printing. Neutral pH collagen could potentially be used for printing trabecular-bone mimicking constructs with cells included directly in the ink, but would require a modified setup with temperature control [35]. Similarly, the SLA setup presents significant challenges for embedding cells in the PEGDA/photoinitiator solution before printing. Photoinitiators type I, along with the free radicals generated upon their activation by light absorption, are inherently toxic to cells [60, 61]. While this toxicity can be mitigated by reducing photoinitiator concentrations and shortening printing times [62], different cell types exhibit varying levels of sensitivity to the generated radicals [61]. Additionally, tailoring the properties of the PEGDA itself, such as using a higher molecular weight PEGDA, may improve cell viability [63].

## Conclusions

In conclusion, collagen scaffolds with trabecular geometries and favorable osteogenic properties were produced using FRESH printing. This high-fidelity 3D printing technique enabled the creation of stable trabecular scaffolds from soft collagen, which would have been impossible with earlier methods. The mechanical properties of the bone-mimicking collagen scaffolds were evaluated together with a thorough analysis of proliferation and osteogenic activity of human osteoblasts and SaOS-2 cells, after seeding on the 3D collagen scaffolds. Our study also included a comparison with PEGDA scaffolds, indicating non-inferiority of the collagen scaffolds, which, given the immunogenicity and other drawbacks of synthetic polymers, encourages further developments of complex printed scaffolds based on natural polymers. Our findings suggest that 3D-printed collagen scaffolds produced using the FRESH technique may offer an attractive alternative to synthetic scaffolds, bringing soft natural polymers closer to applications in bone tissue engineering.

## Electronic supplementary material

Below is the link to the electronic supplementary material.

Supplementary Material 1

Supplementary Material 2

Supplementary Material 3

## Data Availability

The data are available from the corresponding author upon reasonable request.

## Abbreviations

FRESH: Freeform Reversible Embedding of Suspended Hydrogels
PEGDA: Poly(ethylene glycol)-diacrylate
hOBs: Human osteoblasts
SLA: Stereolithography
PLA: Polylactic acid
PDMS: Polydimethylsiloxane
LAP: Lithium phenyl-2,4,6-trimethylbenzoylphosphinate
PEI: Polyetherimide
SEM: Scanning electron microscope
PBS: Phosphate buffered saline
ALP: Alkaline phosphatase
LDH: Lactate dehydrogenase
AR: Alizarin red
DAPI: 4’,6-diamidino- 2-phenylindole
CFDA: Carboxyfluorescein diacetate
OCN: Osteocalcin
