# Supplementary material for: Trabecular-bone mimicking osteoconductive collagen scaffolds: an optimized 3D printing approach using freeform reversible embedding of suspended hydrogels
Source: 3D Print Med. 2025 Mar 11;11:11. doi: 10.1186/s41205-025-00255-0 (PMC11895158; doi:10.1186/s41205-025-00255-0)
Supplement: Supplementary file 3 — Supplementary Material 3 [file 41205_2025_255_MOESM3_ESM.docx]

**Supplementary Figures**

**Trabecular-bone mimicking osteoconductive collagen scaffolds: An optimized 3D printing approach using freeform reversible embedding of suspended hydrogels**


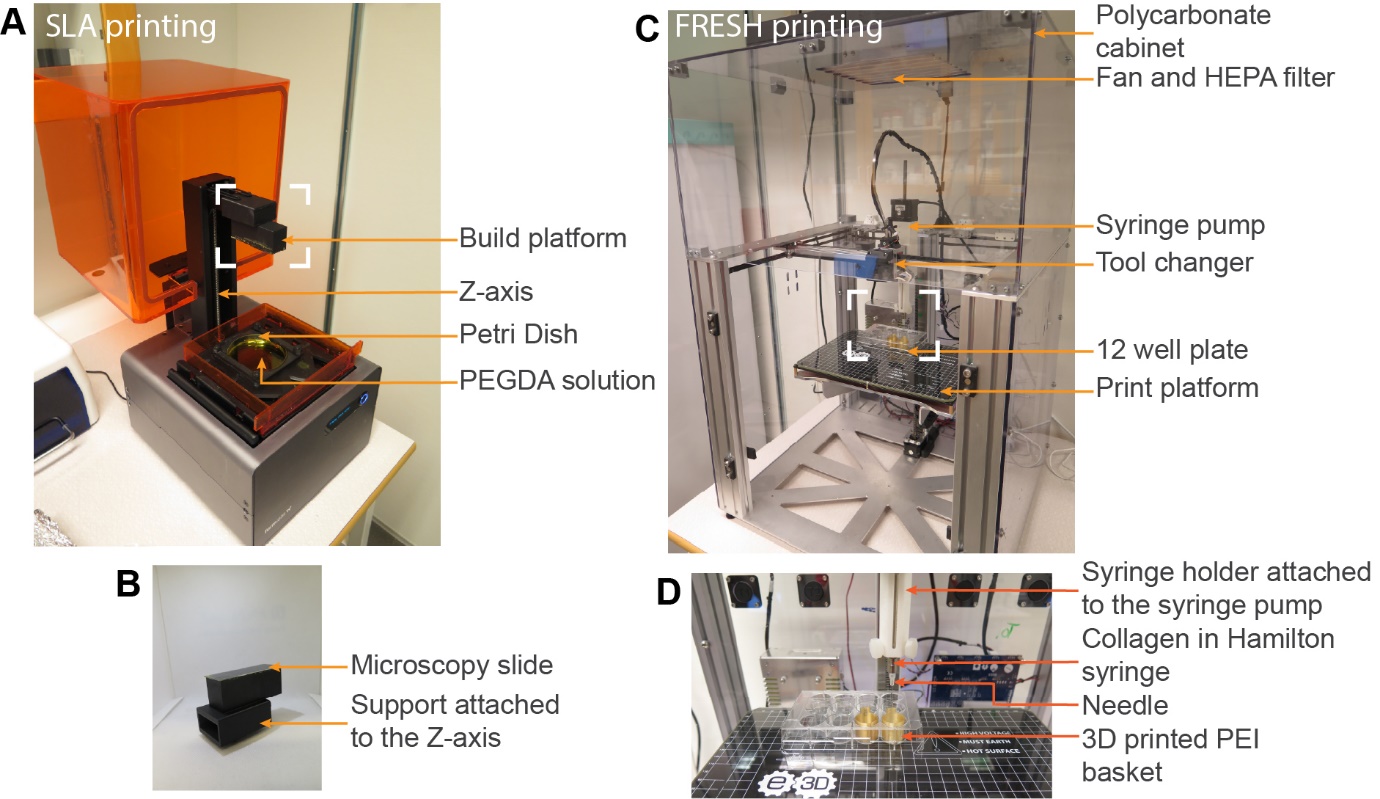


**Supplementary Figure 1.**  Pictures of 3D printing setups for SLA printing (A-B) and FRESH printing (C-D). (A) Form 1+ SLA printer used to fabricate the PEGDA scaffolds. The SLA printing setup features a build platform detailed in (B) attached to the Z axis and a Petri dish serving as the PEDGA solution (yellow) tank. (B) Detail of the custom-made build platform consisting of a microscopy slide glued on a 3D printed support which can be attached to the Z-axis. During the printing process, the build platform moves along the Z-axis, lowering into the PEGDA solution to enable the printing of the scaffold onto the microscopy slide by light exposure with a laser of the photocrosslinkable PEGDA solution. (C) Open source extrusion bioprinter based on the E3D motion system and tool changer as described by Engberg et al (1). The FRESH printing setup features a polycarbonate cabinet with a fan and an HEPA filter for contamination free 3D-printing. The extrusion process is controlled by the syringe pump which is attached to the tool changer during the print. The scaffolds are printed in a 12 well plate placed on the print platform. (D) Detail of the collagen extrusion setup, consisting of a syringe holder attached to the syringe pump. The syringe holder fits a Hamilton syringe, which is fitting a needle and contains acidic collagen. A 12 well plate holds 3D-printed PEI baskets, filled with the support bath. The acidic collagen is printed in the gelatin support bath. The PEI basket are used for easier handling of the FRESH printed scaffolds after gelatin melting.


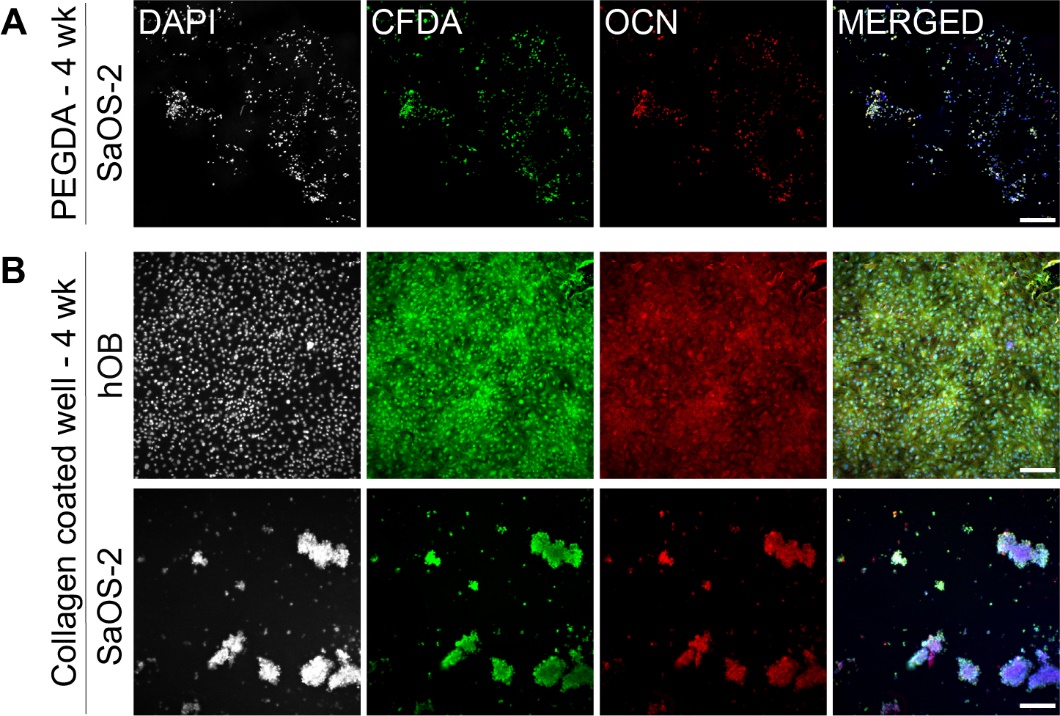


**Supplementary Figure 2.** Immunocytochemistry of osteoblastic cells grown on SLA-printed PEGDA scaffolds and on collagen-coated wells after 4 weeks. Cells were stained for cell nuclei (DAPI), cytosol (carboxyfluorescein diacetate [CFDA]), and the osteoblast marker osteocalcin (OCN). (A) Maximum intensity z projection images captured by confocal microscopy of SaOS-2 cells grown on PEGDA scaffolds after 4 weeks. (B) Image captured by confocal microscope of hOB and SaOS-2 grown on collagen coated wells after 4 weeks. In 2D, hOB form a confluent layer after 4 weeks and SaOS-2 form cell aggregates. Scale bars: 200 μm.

**Reference**

1. Engberg A, Stelzl C, Eriksson O, O’Callaghan P, Kreuger J. An open source extrusion bioprinter based on the E3D motion system and tool changer to enable FRESH and multimaterial bioprinting. Scientific Reports. 2021;11(1).
